# A mutation creating an upstream initiation codon in the *SOX9* 5′ UTR causes acampomelic campomelic dysplasia

**DOI:** 10.1002/mgg3.282

**Published:** 2017-03-21

**Authors:** Anna E. von Bohlen, Johann Böhm, Ramona Pop, Diana S. Johnson, John Tolmie, Ralf Stücker, Deborah Morris‐Rosendahl, Gerd Scherer

**Affiliations:** ^1^ Institute of Human GeneticsUniversity of FreiburgFreiburgGermany; ^2^ Institute of Medical GeneticsYorkhill NHS TrustGlasgowUK; ^3^ Childrens Hospital Hamburg‐AltonaHamburgGermany; ^4^ Present address: Klinik für AnästhesiologieOperative Intensivmedizin und SchmerztherapieUniklinikum Gießen und MarburgGießenGermany; ^5^ Present address: Department of Translational Medicine and NeurogeneticsIGBMCIllkirchFrance; ^6^ Present address: Broad Institute of MIT and HarvardCambridgeMassachusetts02142USA; ^7^ Present address: Harvard Stem Cell InstituteCambridgeMassachusetts02138USA; ^8^ Present address: Department of Stem Cell and Regenerative BiologyHarvard UniversityCambridgeMassachusetts02138USA; ^9^ Present address: Clinical GeneticsSheffield Children's Hospital NHS Foundation TrustSheffieldUK; ^10^ Present address: Clinical Genetics and GenomicsRoyal Brompton and Harefield NHS Foundation Trust and National Heart and Lung InstituteImperial College LondonLondonUK

**Keywords:** Campomelic dysplasia, Kozak consensus, *SOX9*, uAUG, uORF

## Abstract

**Background:**

Campomelic dysplasia (CD) is a semilethal developmental disorder caused by mutations in and around *SOX9*. CD is characterized by multiple skeletal malformations including bending (campomelia) of long bones. Surviving patients frequently have the acampomelic form of CD (ACD).

**Methods:**

This is a single case report on a patient with clinical and radiological features of ACD who has no mutation in the *SOX9* protein‐coding sequence nor a translocation with breakpoint in the *SOX9* regulatory domain. We include functional studies of the novel mutant protein in vitro and in cultured cells.

**Results:**

The patient was found to have a de novo heterozygous mutation c.‐185G>A in the *SOX9* 5′UTR. The mutation creates an upstream translation start codon, uAUG, with a much better fit of its flanking sequence to the Kozak consensus than the wild‐type AUG. By in vitro transcription‐translation and transient transfection into COS‐7 cells, we show that the uAUG leads to translation of a short peptide from a reading frame that terminates just after the wild‐type AUG start codon. This results in reduced translation of the wild‐type protein, compatible with the milder phenotype of the patient.

**Conclusion:**

Findings support the notion that more mildly affected, surviving CD/ACD patients carry mutant *SOX9* alleles with residual expression of SOX9 wild‐type protein. Although rarely described in human genetic disease and for the first time here for CD, mutations creating upstream AUG codons may be more common than generally assumed.

## Introduction

Campomelic dysplasia (CD; MIM #114290) is a semilethal skeletal malformation syndrome characterized by macrocephaly, mid‐face hypoplasia, Robin sequence, hip dislocation, bowed femora and tibiae (i.e., campomelia), talipes, and respiratory distress. Radiological features include hypoplastic scapulae, small chest, 11 rib pairs, undermineralized thoracic pedicles, kyphosis or scoliosis, pelvic malformations, bowing of femora and tibiae, hypoplastic fibulae, and short first metacarpals (Mansour et al. 1995; Unger et al. 2013). About 10% of cases missing the eponymous feature of campomelia are referred to as acampomelic CD (ACD). An additional feature in CD is the XY sex reversal that affects about two‐thirds of karyotypic males.

Campomelic dysplasia has an overall mortality rate of ~75% in neonates, increasing to ~90% by 2 years of age (Mansour et al. 1995). ACD cases appear to have a much better prognosis, with ~65% alive at ≥1 year (Mansour et al. 2002). CD/ACD individuals who survive the neonatal period may have a number of complications, the most common of which include recurrent apnea/respiratory tract infection, progressive kyphoscoliosis, short stature, dislocation of the hips, conductive hearing loss, and mild to moderate learning difficulties (Mansour et al. 2002).

Campomelic dysplasia results from de novo heterozygous *SOX9* mutations. The majority of the mutations are distributed over the entire coding region of the *SOX9* gene and include missense, nonsense, frameshift, and splice mutations, while a few CD cases are due to large deletions covering *SOX9*. As these mutations are predicted to result in loss‐of‐function alleles, CD is regarded as a haploinsufficiency syndrome (Unger et al. 2013). In addition, chromosomal rearrangements including translocations, inversions, and deletions that do not affect the gene body but instead interrupt the *SOX9* regulatory domain, which extends over more than 1 Mb upstream of *SOX9*, can also result in CD of varying severity. In these cases, removal of long‐range tissue‐specific enhancers is assumed to affect *SOX9* expression (Gordon et al. 2009).

## Material and Methods

### Human subjects

This is a single case report. Appropriate informed consent was obtained from the patient's parents.

### Generation of constructs

The 5′UTR of the *SOX9* gene (GenBank NM_000346.3) from wild‐type and mutant DNA was amplified by PCR, cloned into pcR2.1 TOPO (Invitrogen, Karlsruhe, Germany) and subsequently transformed into competent Dh5*α* cells. Plasmids were isolated and forward (5′‐ACT GCT GTG CTG TGA TTG GCG GGT GGC TCT AAG‐3′) and reverse (5′‐CTA GGG CCC TTG GTT GCC CGG GGC CGG GGC AGG GGG CTG G‐3′) primers were used to create a 10 bp frameshift. All constructs, wild type, mutant and frameshift, were cloned in‐frame 5′ to the c‐myc epitope tag using the *HindIII* and *ApaI* site in the pcDNA3.1/myc‐His/B expression vector (Invitrogen). A partial *SOX9* Exon 1‐containing vector (pcDNA3.1/Sox9/1‐304) was digested with *ApaI*, and the resulting 711 bp fragment was introduced into the *ApaI* site next to the epitope of each construct. The constructs were verified by automated sequencing (ABI Prism 3100 Genetic Analyzer, Applied Biosystems).

### In vitro transcription‐translation

For the generation of fusion proteins, the TNT T7 Coupled Reticulocyte Lysate System (Promega, Mannheim, Germany) was used together with ^35^S‐methionine (Amersham, Freiburg, Germany) following the manufacturer's protocol. For this purpose, 1 μg of each construct and 0.5 μg of control plasmid MKKS‐pGBKT7 (encoding a C‐myc‐tagged MKKS protein of 62 kD) were used. After completed TNT reaction, 20 μL of 6 × SDS‐PAGE sample buffer was added to 5 μL of the samples, boiled for 5 min and separated by SDS‐PAGE on a 12% acrylamid gel. The gel was treated with fixation buffer and Amplify Solution (Amersham) and exposed overnight at room temperature using Kodak BioMax MS film (Kodak, Rochester, NY, USA).

### Transfection and immunoblotting

COS‐7 cells were cultured in RPMI supplemented with 10% FCS and 100 μg/mL pen/strep. For expression experiments, approximately 1 × 10^6^ cells were seeded into 100 mm dishes and transfected at 60–80% confluency. Using Lipofectamine™ (Invitrogen), 6 μg of each construct was cotransfected with a control plasmid (4 μg). Each transfection was performed at least in triplicate and with two different DNA preparations of each construct. Cells were harvested 24 h posttransfection, lysed in 300 μL RIPA buffer, sonified and centrifuged at 10000 rpm for 1 min. Cell lysates were resolved by SDS‐PAGE on a 12% acrylamide gel and analyzed by immunoblotting using primary mouse anti‐c‐myc‐antibody (1:1000, BD Bioscience, Heidelberg, Germany) at 4°C over night, followed by a secondary antibody (1:5000) for 1 h at RT. The signals were monitored by incubating with ECL reagent (Amersham) for 1 min. A Fuji Medical Super RX X ray film (Fuji, Düsseldorf, Germany) was exposed according to the signal intensity.

## Results

### Clinical course and diagnostic workup of patient

The patient and her healthy twin brother were born at 40 weeks gestation by cesarean section. Parents are healthy and unrelated and conceived the twins by IVF. Apgar scores for the patient were 1^9^ and 5^9^. Birth weight was 2.44 kg (<2nd centile) and OFC 35.4 cm (50th–98th centile). She had a dusky episode at 10 minutes which resolved with oxygen but showed increasing respiratory distress requiring intubation and ventilation by day 3. A tracheostomy tube was inserted because of persisting requirements for ventilation. Bronchoscopy showed tracheomalacia with extrinsic compression. An aortopexy was performed to relieve compression caused by the right brachiocephalic artery. She required gastrostomy because of failure to thrive.

**Figure 1:**
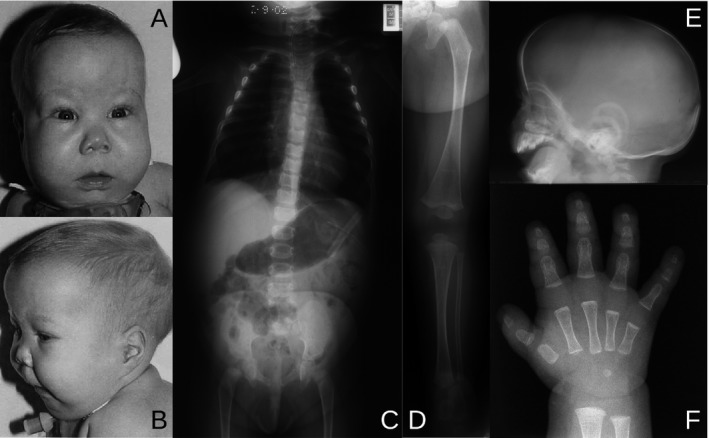
Clinical and radiological features of the patient at 7 months of age. (A, B) Facies with flat nasal root, epicanthic folds, long philtrum, and micrognathia; macrocephaly and low‐set ears. (C–F) Skeletal survey. (C) Hypoplastic scapulae, cervical kyphosis, thoracic scoliosis with undermineralized thoracic pedicles, 11 pairs of ribs, short ischia and unossified inferior pubic rami. (D) Lower limb with straight femur, tibia, and fibula, with delayed ossification of the femoral epiphysis. (E) Lateral skull film showing enlargement of the cranial vault to the size of the facial bones. (F) Hand with short metacarpal I and moderately short middle and distal phalanges.

Referral was made to the genetics department at Glasgow at age 7 months. On examination, she had macrocephaly, a flat nasal root, epicanthic folds, a long philtrum, micrognathia, and low‐set ears (Fig. 1A,B). Skeletal survey revealed hypoplastic scapulae and cervical kyphosis, thoracic scoliosis with undermineralized thoracic pedicles, 11 rib pairs, very short ischia, and unossified inferior pubic rami (Fig. 1C); straight femora, tibiae, and fibulae with delayed ossification of the femoral epiphyses (Fig. 1D); enlarged cranial vault (Fig. 1E); and short metacarpal I and moderately short middle and distal phalanges (Fig. 1F). She had a dilated left renal pelvis, a small patent foramen ovale and mild sensorineural hearing loss. Karyotype is 46,XX. The patient therefore has 3/5 radiological and 5/12 clinical features used as diagnostic criteria for CD (Mansour et al. 1995). A clinical diagnosis of acampomelic campomelic dysplasia, ACD, was made.

To alleviate problems with respiration, VEPTR (vertical expandable prosthetic titanium rib) implants were applied at age of 5 years, which were subsequently expanded by repeated operations. At age 12, a vertebral column resection with removal of two apical vertebrae, anterior column support and long posterior fusion from the second thoracic to the third lumbar vertebra was performed. About 2 years later, she developed late infection and implant removal was required. She carried a tracheostomy tube and needed assisted ventilation at night until age 14, when the tracheostoma was successfully closed. At her present age of 15 years, she still has hearing problems and requires tympanostomy tubes, needs orthopedic shoes, and has a strong wheat allergy. She attends an international school, where she performs rather well.

### Molecular genetic results

**Figure 2:**
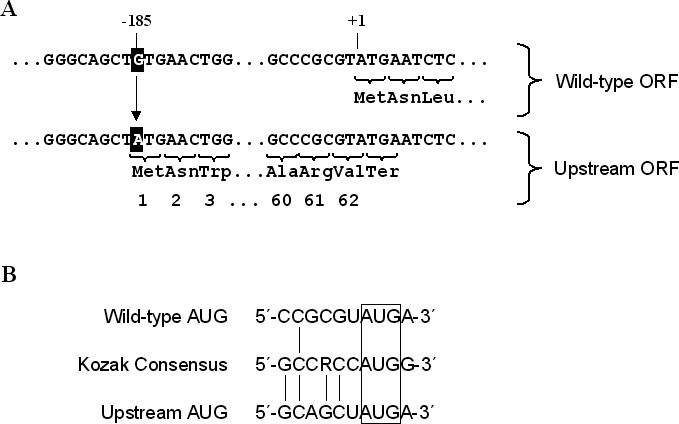
Point mutation in the *SOX9* 5′UTR creates a start codon. (A) The heterozygous G>A transition at position –185 in the *SOX9* 5′UTR in the patient results in an ATG codon that generates an upstream open reading frame (ORF) of 62 codons in a + 1 frame relative to the wild‐type ORF, terminating one nucleotide beyond the normal ATG start codon. (B) The sequence flanking the mutant upstream AUG has a better fit to the Kozak consensus sequence as the sequence around the wild‐type AUG.

As sequence analysis of the *SOX9* coding region and exon/intron boundaries failed to reveal a mutation in the patient, the 372 bp 5′ untranslated region (UTR) was scrutinized following amplification with forward (5′‐ACT GCT GTG CTG TGA TTG GCG‐3′) and reverse (5′‐CTT CTC CTG CTC GTC GGT CAT C‐3′) primers. A heterozygous mutation c.‐185G>A in the 5′ UTR was found. This sequence alteration gives rise to a novel upstream AUG (uAUG) translation initiation codon 185 nucleotides before the normal AUG start codon (the A corresponding to nucleotide +1). The uAUG creates a novel upstream open reading frame (uORF) of 62 codons which is out‐of‐frame with the normal ORF and which terminates just after the wild‐type start codon (Fig. 2A). The patient's parents and the healthy twin brother were homozygous G/G at this position, indicating a de novo mutation in the patient. The G>A transition is not found in 100 control chromosomes, dbSNP, or the ExAC consortium database (Lek et al. 2016) nor in 30 CD or CD‐like cases without a *SOX9* coding region mutation. Paternity was confirmed by microsatellite marker analysis. Interestingly, the flanking sequence of the upstream AUG translation start codon matches the Kozak consensus (Kozak 1986) much better than the sequence preceding the wild‐type AUG start codon (4/7 vs. 1/7) (Fig. 2B).

### Expression studies

**Figure 3:**
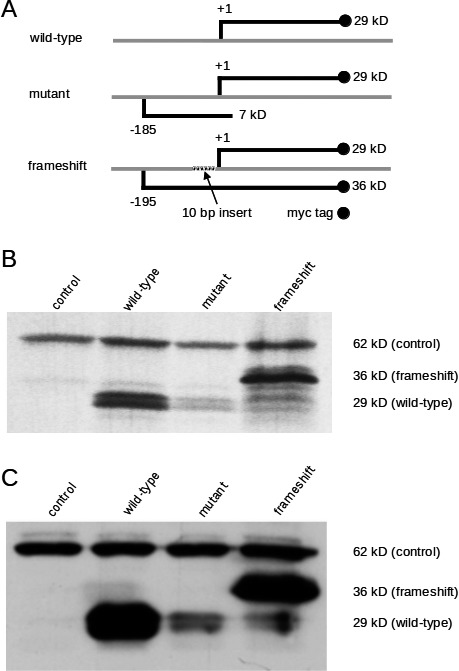
Preferential translation initiation from the mutant AUG reduces expression from the normal AUG start codon. (A) All expression constructs were derived from pcDNA3.1/myc‐His/B and contain the T4 bacteriophage T7 promoter and the mammalian cytomegalovirus (CMV) promoter driving expression of the partial *SOX9* mRNA. The wild‐type construct contains the native *SOX9* 5′UTR, whereas the mutant construct contains both the wild‐type and the mutant AUG. Both constructs are predicted to express a partial *SOX*9 protein of 29 kD with a myc‐tag at the C terminus, whereas the mutant construct expresses a 7 kD peptide lacking the epitope tag. The frameshift construct with a 10 bp insert encodes two epitope‐tagged proteins, a 36 kD protein initiated from the mutant AUG, and the 29 kD partial SOX9 protein initiated from the normal AUG. (B) Transcription‐translation of the indicated constructs was performed using a T7‐coupled rabbit reticulocyte lysate system. The wild‐type construct was adequately expressed, while only a weak signal could be detected in case of the mutant construct. The frameshift construct allowed the expression of two tagged proteins. Note that the initiation from the novel AUG is more intense than from the native AUG. (C) The identical constructs were transfected into COS‐7 cells and expression was monitored by western blotting. Lysates of cells transfected with the wild‐type or mutant construct demonstrated strong or weak translation initiation, respectively, from the normal start codon. Transfection of the frameshift construct revealed preferential initiation from the novel AUG. In (B) and (C), plasmid MKKS‐pGBKT7 encoding a 62 kD c‐myc‐tagged protein was used as control.

To analyze the translation initiation potential of the upstream AUG, we generated c‐myc‐epitope‐tagged partial SOX9 expression constructs in pcDNA3.1/myc‐His/B bearing either wild‐type or mutant 5′‐UTR sequences. Adopting a previously described strategy (Liu et al. 1999), a frameshift construct with a 10 bp insert within the upstream ORF was also made to obtain an in‐frame peptide initiating at the uAUG and extending up to the c‐myc tag. The frameshift construct allowed us to evaluate the translational activity at the mutant and the wild‐type start codon. Conceptual translation from the wild‐type construct results in a 29 kD protein with the c‐myc‐tag, whereas translation from the mutant construct yields the 29 kD wild‐type protein and a 7 kD protein lacking the epitope tag. Translation from the frameshift construct predicts the wild‐type protein as well as a novel, longer 36 kD protein, both with the epitope tag (Fig. 3A).

In vitro transcription‐translation confirmed that the wild‐type construct directed expression of the 29 kD protein. The mutant construct yields less protein, indicating a residual translational activity at the wild‐type start codon. The frameshift construct drives translation of both the 29 kD wild‐type and the 36 kD novel protein. The stronger signal corresponding to the novel protein indicates preferential translation from the mutant AUG (Fig. 3B).

To confirm that the mutant AUG directs translation also in eukaryotic cells, the same constructs were transfected into COS‐7 cells, and expression was monitored by western blot analysis with an anti‐c‐myc‐antibody. The results (Fig. 3C) were in complete agreement with those obtained by in vitro transcription‐translation shown in Fig. 3B. As internal control, a construct driving expression of a 62 kD c‐myc‐tagged MKKS protein was used (Fig. 3B,C).

We conclude that the c.‐185G>A mutation has functional consequences for translation initiation at the normal AUG in vitro, and by extrapolation reduces expression of the native SOX9 protein in normal tissues.

## Discussion

As shown by in vitro transcription‐translation and transient transfection assays, the de novo mutation in the *SOX9* 5′ UTR results in preferential initiation of translation at the uAUG at the expense of efficient translation initiation at the wild‐type AUG start codon. The fact that generation of wild‐type SOX9 protein from the mutant allele of this ACD patient is not entirely abolished indicates that the situation is not complete haploinsufficiency. A genotype/phenotype correlation has repeatedly been postulated based on the observation that mutant *SOX9* alleles with supposedly residual function lead to the milder, acampomelic form of CD (Scherer et al. 2000; Staffler et al. 2010). This assumption is strengthened by the fact that ACD cases are overrepresented in the group of *SOX9* translocation cases and in the group with *SOX9* missense mutations. In both groups, the affected *SOX9* alleles are not obvious loss‐of‐function alleles and thus can be assumed to retain residual activity. Two recent publications provide comprehensive lists of *SOX9* mutations in CD/ACD. The first lists about 70 mutations in the *SOX9* coding region, of which 11 are from ACD cases, almost exclusively missense mutation in the DNA‐binding HMG domain of the SOX9 transcription factor (Table S3; Mattos et al. 2015). The other report summarizes chromosomal rearrangements upstream and downstream of *SOX9*, with 13 and nine rearrangements reported for ACD and CD cases, respectively (Table 1; Fonseca et al. 2013). Three additional ACD mutations have meanwhile been published, two missense mutations (Gopakumar et al. 2014; Preiksaitiene et al. 2016) and one translocation (Walters‐Sen et al. 2014). Despite this large number of reported *SOX9* mutations in ACD, the functional consequences of these mutations have only occasionally been tested experimentally. For five missense mutations, four in the HMG domain (M113V, R152P, H165Q, and H169Q) and one in the dimerization domain (A76E), residual DNA‐binding (Meyer et al. 1997) and also transactivation of SOX9 target genes has been demonstrated (Sock et al. 2003; Staffler et al. 2010; Matsushita et al. 2013). Pop et al. (2005) studied a cohort of 10 CD cases, including several cases with longer survival and two ACD cases, which were all heterozygous for the nonsense mutation Y440X that truncates the C‐terminal transactivation domain spanning residues 402–509 of SOX9. Cell transfection experiments revealed that the mutant SOX9 protein retained some transactivation activity on SOX9 target genes (Pop et al. 2005). In addition, in a t(13;17) ACD translocation case surviving for at least 26 years (case 2 in Tommerup et al. 1993), expression of both *SOX9* alleles has been demonstrated in lymphoblasts from the patient (Wirth et al. 1996). The documentation that some protein is made from the normal ORF of the mutant *SOX9* allele in our patient gives further support to the notion that more mildly affected, surviving CD/ACD patients like the present case may carry mutant *SOX9* alleles which are hypomorphic rather than complete null alleles.

The G>A mutation creates an AUG triplet in the 362 nucleotide *SOX9* 5′ UTR that is normally devoid of AUGs. That this mutant upstream AUG triplet functions as an effective initiator codon results from two facts. Firstly, mRNAs are usually translated by a scanning mechanism, whereby the ribosome enters at the capped 5′ end of the mRNA and migrates linearly until it encounters the first AUG codon, which is the uAUG in the present case. Secondly, the uAUG triplet resides in a sequence context that is more favorable for translation initiation than that at the wild‐type AUG. While the latter matches the GCCRCCAUGG Kozak consensus only at one position, the sequence around the uAUG matches it at four positions. The purine (R) 3 nucleotides before the AUG in the Kozak sequence and the G following the AUG make the strongest contributions to an optimal translation initiation (Kozak 1986, 1997). Interestingly, the uAUG has a G residue 3 nucleotides before the AUG, while the normal AUG has a C at this position; in either case, an A follows the AUG. Although the uAUG is the initiation codon closest to the 5′ end of the mRNA, some translation still occurs at the wild‐type AUG. This possibly results from a reinitiation mechanism, which allows access to an internal start codon. Because reinitiation occurs only in the forward direction, the strongest constraint is imposed by an upstream ORF that overlaps the internal start site leading to low‐level translation (Kozak 2002), as in the present case. The second possibility is leaky scanning, whereby ribosomes bypass the first AUG and initiate translation farther downstream, usually when the first AUG codon resides in a suboptimal context (Kozak 2002). Although the sequence at the mutant uAUG has a better match to the Kozak consensus than the wild‐type AUG, it is still suboptimal, as it has an A and not a G residue following the AUG. Thus, either or both of these possible mechanisms may be responsible for the observed low‐level initiation at the *SOX9* start codon.

A mutation in the 5′UTR creating a functional uAUG codon has first been described in autosomal dominant hereditary melanoma. There, a novel uAUG in *CDKN2A* led to decreased translation from the wild‐type AUG and to predisposition to melanoma. As in our case, the short out‐of‐frame ORF initiated from the uAUG overlaps the *CDKN2A* start codon and resulted in profound inhibition of translation initiation at the normal AUG (Liu et al. 1999). Mutations creating uAUGs have subsequently been described and functionally tested in 12 other human disorders (reviewed by Barbosa et al. 2013). In all these cases, the uORFs generated by the novel uAUGs reduced protein expression from the normal ORF to 30% or less. Two further examples of novel uAUGs compromising translation at the normal start site were reported: in the *IFITM5* gene in osteogenesis imperfecta (Cho et al. 2012; Semler et al. 2012), and in the *GRHPR* gene in primary hyperoxaluria type II (Fu et al. 2015). Together with these reports, our study emphasizes that for mutation screening in human disorders, the 5′ UTR should be routinely analyzed for potentially pathogenic variants generating novel uAUGs and reading frames.

In conclusion, we report the first CD/ACD case with a de novo base substitution in the 5′ UTR of *SOX9* that generates a functional upstream start codon and an upstream ORF that is out‐of‐frame with the main coding sequence, leading to reduced but not completely abolished expression of the normal ORF. Our study corroborates the notion that more mildly affected surviving CD/ACD patients carry mutant *SOX9* alleles with residual expression of SOX9 wild‐type protein.

## Conflict of Interest

None declared.
